# CodingQuarry: highly accurate hidden Markov model gene prediction in fungal genomes using RNA-seq transcripts

**DOI:** 10.1186/s12864-015-1344-4

**Published:** 2015-03-11

**Authors:** Alison C Testa, James K Hane, Simon R Ellwood, Richard P Oliver

**Affiliations:** Centre for Crop and Disease Management, Department of Environment and Agriculture, School of Science, Curtin University, Bentley, WA 6102 Australia; Postal address: Department of Environment and Agriculture Centre for Crop and Disease Management, GPO Box U1987, Perth, 6845 Western Australia

**Keywords:** Generalised hidden Markov model, Gene annotation, Fungi, Gene prediction

## Abstract

**Background:**

The impact of gene annotation quality on functional and comparative genomics makes gene prediction an important process, particularly in non-model species, including many fungi. Sets of homologous protein sequences are rarely complete with respect to the fungal species of interest and are often small or unreliable, especially when closely related species have not been sequenced or annotated in detail. In these cases, protein homology-based evidence fails to correctly annotate many genes, or significantly improve *ab initio* predictions. Generalised hidden Markov models (GHMM) have proven to be invaluable tools in gene annotation and, recently, RNA-seq has emerged as a cost-effective means to significantly improve the quality of automated gene annotation. As these methods do not require sets of homologous proteins, improving gene prediction from these resources is of benefit to fungal researchers. While many pipelines now incorporate RNA-seq data in training GHMMs, there has been relatively little investigation into additionally combining RNA-seq data at the point of prediction, and room for improvement in this area motivates this study.

**Results:**

CodingQuarry is a highly accurate, self-training GHMM fungal gene predictor designed to work with assembled, aligned RNA-seq transcripts. RNA-seq data informs annotations both during gene-model training and in prediction. Our approach capitalises on the high quality of fungal transcript assemblies by incorporating predictions made directly from transcript sequences. Correct predictions are made despite transcript assembly problems, including those caused by overlap between the transcripts of adjacent gene loci.

Stringent benchmarking against high-confidence annotation subsets showed CodingQuarry predicted 91.3% of *Schizosaccharomyces pombe* genes and 90.4% of *Saccharomyces cerevisiae* genes perfectly. These results are 4-5% better than those of AUGUSTUS, the next best performing RNA-seq driven gene predictor tested. Comparisons against whole genome *Sc. pombe* and *S. cerevisiae* annotations further substantiate a 4-5% improvement in the number of correctly predicted genes.

**Conclusions:**

We demonstrate the success of a novel method of incorporating RNA-seq data into GHMM fungal gene prediction. This shows that a high quality annotation can be achieved without relying on protein homology or a training set of genes. CodingQuarry is freely available (https://sourceforge.net/projects/codingquarry/), and suitable for incorporation into genome annotation pipelines.

## Background

Whole-genome sequencing has enabled investigations into the gene content of living many organisms and forms the foundation for further study of gene expression, proteomics and epigenetics. After assembly of a novel genome, gene annotation is often the first step in analysing the gene content of an organism. Accurate annotation of the exonic structure of genes is crucial to the success of all subsequent functional and comparative analyses.

Problems that can potentially be caused by incorrect gene annotation are numerous and can lead to incorrect assessments of the lifestyle and ecology of an organism. In comparative genomics where orthologous genes or conserved functional domains are compared between species/isolates, the estimated numbers of such genes/domains can be distorted by less than perfect annotations (as described by Hane et al*.* [1], S Text 1). Prediction of extracellular secretion, which can be determined by a short signal peptide at the N-terminus, can miss secreted proteins if the start codon of a gene has been incorrectly annotated. Mis-annotating the start of protein translation could either cut off the signal peptide or bury it within the annotation. While a seemingly benign annotation error, the consequences for downstream research could be detrimental, particularly as the biotic interactions or industrial applications of microbes are largely determined by their secretomes. Additionally, translated protein sequences of novel species are often submitted to databases such as NCBI [2] and Uniprot [3]. It is commonplace to use these database entries to support the annotation of related species or isolates, meaning errors present in the pioneer annotation may be repeated. When these new annotations based on false assumptions are added to databases, there is not only a propagation of errors, but also a perceived strengthening of homology evidence for incorrect protein sequences.

In recent years, correction of *in silico* predicted gene annotations with RNA-seq derived transcripts and read alignments has enabled vastly improved genome annotations and corrections of annotated gene structures [4-6]. Short read and/or assembled transcript alignments are typically used to correct the coordinates of intron-exon boundaries in existing gene annotations or predictions [7], to train gene predictors [8], and can also be incorporated directly into gene prediction by hybrid gene predictors [9,10]. Since their initial application to gene prediction [11], generalised hidden Markov models (GHMMs) have played an important role in genome annotation. Various GHMM gene predictors [12-15] continue to be incorporated into annotation pipelines [16-18], some of which are capable of making use of RNA-seq data. For example, AUGUSTUS [9,10,14] allows the user to generate hint files from RNA-seq read/transcript alignments that are then used to improve prediction accuracy. More recently, a new version of GeneMark-ES [15], named GeneMark-ET [8] allows the incorporation of RNA-seq data into its automated gene model training. These gene finders are both applicable to a broad range of eukaryotic genomes. A number of pipelines have also been developed that utilise available gene prediction software and RNA-seq data to generate annotations. Some examples of such pipelines are Maker [16,19], EVidenceModeler [7], JAMg [20], SnowyOwl [18] and the insect genome annotation pipeline OMIGA [21]. The continued development of pipelines such as these relies on the availability and development of component software such as GHMM gene predictors.

Fungal genomics has applications in areas such as agriculture [22-24], medicine [25,26], biomass conversion [27,28] and food/beverage production [29,30]. This broad industry relevance and the continued growth in the number of new fungal species with sequenced genomes emphasises the importance of fungal gene annotation. Fungal genomes differ from those higher eukaryotes in that they are gene dense with short introns [31,32]. They also exhibit less alternate splicing when compared to other eukaryotes, with a higher proportion of mRNA isoforms arising from retained introns [33]. Manual annotation is considered to be the most reliable method of producing a high quality genome annotation, but this is time consuming and can be a bottleneck in genome studies [34]. Consequently fungal genome annotations are typically derived from *ab initio* predictions, spliced EST/transcript alignments and protein homology [34]. For many fungi, closely related species have either not been sequenced or their genomes have not been annotated in detail. This can mean that sets of homologous proteins for use in protein homology annotation are either small or unreliable. In such cases, gene prediction relies more on EST/transcript alignments and *ab initio* predictions.

Currently available gene prediction software and pipelines are typically intended for application across a broad range of eukaryotes, with comparatively few being specific to fungi. GipsyGene [35] is a GHMM gene predictor that was developed for fungi, with particular attention given to modelling fungal introns correctly. A version of GeneMark-ES [15], a self-training GHMM, also uses an intron model designed for fungi. However, neither of these incorporates RNA-seq data. SnowyOwl [18] is a recently developed pipeline designed specifically to annotate fungal genomes using RNA-seq data and homology information. Although designed for fungi, SnowyOwl selects from GHMM predictions made by AUGUSTUS [9,10,14], a gene predictor that was optimised for application across a broad range of eukaryotes.

In this study we present the gene prediction tool CodingQuarry. It is designed to make protein-coding gene sequence predictions through the use of assembled or aligned RNA-seq transcripts in both GHMM training and prediction. CodingQuarry is differentiated from other gene predictors by the combined use of gene predictions made directly from both transcript and genome sequences.

The choice to tailor CodingQuarry to the prediction of fungal genes and to use assembled, aligned transcripts rather than raw read alignments relates to some key differences between fungal genomes and those of higher eukaryotes. Firstly, fungi exhibit significantly less alternative splicing than higher eukaryotes. Consequently, the task of transcript assembly is simpler, resulting in a higher proportion of correctly assembled full-length transcripts [36]. Secondly, fungi have smaller introns than higher eukaryotes [32]. Recent studies indicate short introns are reconstructed in transcript assembly with a higher success rate than long introns [37]. These transcript assembly advantages make it feasible to generate coding sequence annotations directly from assembled transcript sequences, a process that is more likely to be error prone in higher eukaryotes.

A major consequence of the high gene density observed in fungi is a high proportion of instances whereby the untranslated regions (UTRs) of adjacent transcripts overlap in terms of their positions on genomic DNA. Overlap can be between 3′ and 5′ UTRs of adjacent genes on the same strand, or between 5′ and 5′ or 3′ and 3′ UTRs of adjacent genes on opposite strands. Overlaps from the latter example, particularly in the case of 3′ to 3′, are referred to as sense-antisense (S-AS) overlaps. S-AS overlaps have been observed to occur rarely in many species, but are widespread in fungi [38,39]. Essentially this means that in gene-dense fungal genomes, mapped RNA-seq reads belonging to adjacent genes may support regions of coverage that span two or more loci. This is a more severe problem when ‘unstranded’ RNA-seq chemistries are used, as S-AS overlaps can be distinguished through the use of stranded RNA-seq data. CodingQuarry is designed to work with assembled, aligned transcripts derived from either stranded or unstranded RNA-seq data and to specifically address the problem of merged transcripts, such that these transcript assembly errors do not translate to coding sequence annotation errors or omitted gene loci.

For the purpose of demonstrating CodingQuarry’s performance we have selected two exemplar fungal species, which possess highly reliable sequence and annotation resources: *Saccharomyces cerevisiae* and *Schizosaccharomyces pombe. S. cerevisiae*, commonly known as Baker’s yeast, has long been a model organism and is important to the wine making, baking and brewing industries. *Sc. pombe*, commonly known as fission yeast, is also a model organism. These two species are estimated to have diverged from a common ancestor up to 1000 million years ago [40,41] and are representative of distantly related fungal sub-phyla. In this study we have used the high-quality annotations of these fungi to benchmark the sensitivity and specificity of CodingQuarry, and compare it to other gene predictors.

## Implementation

### Data sets for benchmarking

To test the accuracy of predictions made by CodingQuarry and other gene predictors, we utilised assembled genome sequences, RNA-seq reads and up-to-date gene annotations of two model fungi: *S. cerevisiae* and *Sc. pombe*.

The *Sc. pombe* (isolate 972h-) genome, annotation and protein sequences were downloaded from PomBase [42] and RNA-seq reads [SRA: SRX040571] were downloaded from NCBI [43]. The reads were trimmed using Cutadapt [44], aligned to the genome using TopHat [45,46] (version 2.0.19, −-mate-inner-dist 280, −-mate-std-dev 70, −-min-intron-length 10, −-max-intron-length 5000, −-min-segment-intron 10, −-max-segment-intron 5000) and assembled using Cufflinks [47] (version 2.1.1, −-min-intron-length 10, −max-intron-length 5000, −-overlap-radius 10, −-min-isoform-fraction 0.4, −-library-type fr-firststrand). The RNA-seq data used for *Sc. Pombe* was stranded (i.e. the strand of genomic DNA that produced the mRNA fragment is known). To simulate a transcript assembly from unstranded RNA-seq data, TopHat and Cufflinks were also re-run as above with the modified parameter ‘–library-type fr-unstranded’.

The *S. cerevisiae* (isolate S288c) genome, annotation and protein sequences were downloaded from the Saccharomyces Genome Database [48] and RNA-seq reads [SRA: SRR1198662-8] were downloaded from NCBI. Reads were trimmed, aligned and assembled using the same method as described above for *Sc. pombe* (stranded only, −-mate-inner-dist 200, −-mate-std-dev 40).

Although both *Sc. pombe* and *S. cerevisiae* are annotated to a high standard, it was desirable to identify a stringent subset of their genes that are of very high-confidence. This is because not all genes are verified to the same degree, and some are therefore more likely to be accurate than others. It is still possible that the full annotations contain errors that are artefacts of the prediction tools, data and methods used to generate them. Comparing predictions against a high-confidence set excludes some annotations that are lower confidence, and is likely to give a better assessment of the accuracy of gene predictors. Annotations within these high-confidence subsets were required to exactly match sequences in Uniprot’s [3] reviewed database and to be listed and as possessing protein level evidence. There were 1,898 of these for *Sc. pombe* and 5,224 for *S. cerevisiae*. Nevertheless, as CodingQuarry’s intended purpose is to predict genes across entire fungal genomes, we also report its performance benchmarked to the less stringent full datasets of 5,124 *Sc. pombe* genes and 6,575 *S. cerevisiae* genes.

### CodingQuarry prediction method outline

CodingQuarry predicts genes in 2 stages. The first stage involves prediction of genes directly from transcript sequences derived from regions of the genome supported by RNA-seq in GFF (General Feature Format) [49], such as derived from Cufflinks [47]. The second stage complements the first and involves additional predictions based on genomic sequences. In both stages GHMMs are used to predict genes, however, these differ in their structure and in how they incorporate RNA-seq data into their predictions. The GHMMs used in both stages are also trained automatically using the RNA-seq data. The final predicted annotation produced by CodingQuarry is a combination of predictions made in stages 1 and 2.

#### Stage 1: Training and prediction from transcript sequences

The coordinates of transcribed regions (in GFF format) relative to the assembled genome sequence are used to extract the sequences of a set of virtually spliced transcripts (i.e. intron sequences are removed). A generalised hidden Markov model (GHMM) is used to make gene predictions directly from this set of transcript sequences. Predicted coding-sequences are then converted back to their relative genomic coordinates, with transcript splicing being accounted for in this process.

**Figure 1:**
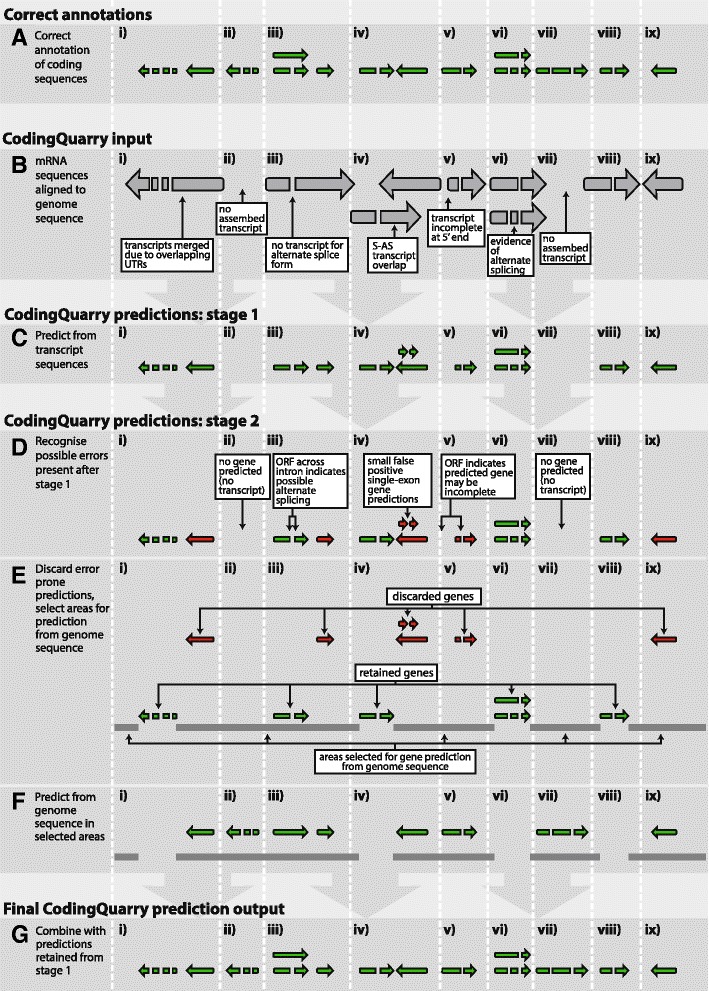
**CodingQuarry flow diagram.** Examples are shown of correct annotations of coding sequences, **(A)** and a typical CodingQuarry input; assembled transcripts aligned to the genome **(B)**. The stages used within CodingQuarry to predict coding sequences are shown **(C-G)**. Firstly, coding sequences are predicted from transcript sequences (introns are removed) using a GHMM **(C)**. Possible prediction errors after this step are coloured red, and notes show how these are identified **(D)**. These error prone predicted genes are discarded **(E)**, and regions are selected for prediction from genome sequence **(F)**. The resulting prediction is output by CodingQuarry **(G)**, which merges the retained predictions from transcript sequences **(E)** with the predictions from selected areas of the genome sequence **(F)**. Sections of the example genome sequence and annotations have been labelled **i-x** in each part of the diagram **(A-G)**, and marked with vertical dotted lines. These sections are labelled to facilitate in-text references to the diagram in the Implementation section of this manuscript. Labels **i-x** correspond to the same genome sections through **A-G**.

The GHMM used in stage 1 uses fixed length states to describe the gene start and Kozak sequence [50] and gene stop codon, and variable length states to describe gene coding sequences, UTRs, and non-coding transcripts. To address the issue of merged transcripts, this model allows a single transcript sequence to contain multiple genes, via the creation of a “middle UTR” state. Where UTRs of adjacent transcripts overlap in terms of their relative corresponding positions on the genomic DNA, a single transcript sequence as derived from RNA-seq can contain multiple gene loci. A pictorial example of this is shown in Figure 1, section Bi, in which the middle UTR state is used to allow the correct prediction of two genes on the same strand within a merged transcript sequence. In the case of unstranded RNA-seq, prediction errors arising from transcript sequences merged due to S-AS UTR overlap are corrected in stage 2.

The coding regions are modelled using a fifth-order, three-periodic Markov chain. The 5′, 3′ and ‘middle’ UTRs, as well as non-coding transcripts are modelled using a fifth-order (non-periodic) Markov chain. A second-order weighted array matrix over a region of 11 nucleotides up to and including the ATG start codon models the Kozak sequence and gene start. Length distributions of the coding region state, UTR states and non-coding transcript state are modelled using smoothed length frequencies.

A self-training method is used, where parameters are initially estimated from the longest open reading frame (beginning with a methionine) in each transcript. The GHMM is then successively run and retrained twice to refine the parameters. There are some restrictions placed on the sequences that are used for retraining, based on the general principle of preferential exclusion of some correct sequences rather than risking including false-positives. Training of the “gene” state is therefore restricted to coding sequence lengths greater than or equal to 600 nucleotides to guard against the inclusion of false-positive predictions in the training set. Similarly, open reading frames in UTR regions greater than or equal to 300 nucleotides are removed from the UTR training set to guard against the inclusion of coding sequences. Where there are overlapping genes in the prediction, the longer gene is retained in the training set and the shorter overlapping gene(s) are discarded.

Importantly, this method is distinct from methods where the transcript/EST alignment is used to inform a GHMM prediction from genome sequence. The main advantage of the initial prediction from transcript sequences is that the predicted annotation will have intron boundaries that agree exactly with the intron boundaries in the transcripts to genome alignment. Another advantage is that where the transcript assembly indicates that there is an alternative splicing, prediction from transcripts allows the coding sequences splicing alternatives to be predicted.

#### Stage 2: Prediction from the genome sequence

After the prediction from transcript sequences is carried out in stage 1, there may still be a number of errors and omissions in the predicted annotation (see Figure 1, section D). These predictions are therefore added to, and in some cases replaced by predictions made from genome sequence.

The stage 1 predicted gene set is used to train a second, different GHMM which is designed to make predictions from assembled genome sequence. This genome based GHMM includes additional states to model introns, a feature not previously required in the spliced transcript-based GHMM used in stage 1. Another difference is that the GHMM used for transcript sequences models the 5′ and 3′ UTR regions, whereas the GHMM used for prediction from genome sequence models these regions as part of larger “intergenic” regions. The stage 2 GHMM intron model used has fixed length states for the donor, acceptor and branch point sequences, modelled by first-order weighted array matrices. Variable length states are used to model the regions between these fixed length states. The intron model is based on research showing that fungal introns have high information content in the 5′ splice site, 3′ splice site and branch point regions [32], and is similar to the intron model used by GeneMark-ES [15]. During training, the acceptor/donor lengths are automatically adjusted by CodingQuarry to suit the fungi being predicted on. The acceptor/donor is extended to the furthest out nucleotide position (up to a maximum length) with a statistically significant difference in nucleotide composition when compared to the adjacent intron region. A Chi-square test (p-value 0.01) is used to test for statistical significance. The acceptor and donor are taken to extend 2 nucleotides into the adjacent exon, and can be up to a maximum length of 22 nucleotides. During prediction, the lengths of these states is fixed. The maximum intron size is set as 10% longer than the longest intron evidenced by the transcript alignment, unless this value is greater than 5,000, in which case the maximum is limited to 5,000. The user can choose to disable the intron model length restrictions of CodingQuarry in order to allow it to be used for species with longer intron lengths.

In prediction from transcript sequences (stage 1), the location of introns is inferred from the transcript to genome alignment, and the assembled transcript sequences are used to model the UTRs. When predicting genes from genome sequence in stage 2, RNA-seq data is also incorporated in GHMM prediction, but in a different way. Where there is supporting evidence from RNA-seq data, the prediction of introns is restricted by the transcript alignment. Intron boundaries (donor and acceptor sites) are disallowed in areas where there is an aligned transcript sequence on the same strand. This restriction is relaxed within 50 nucleotides of the transcript end, where introns may be predicted *ab initio*, in the same manner as in regions without evidence of transcription. Introns are only allowed to occur where the first 2 nucleotides of the intron donor and last two nucleotides of the intron acceptor sites are GT and AG respectively.

After the stage 1 genes are used for training, certain predicted genes that are likely to be inaccurate are discarded and areas of the genome are selected for prediction from genome sequence. Discarded stage 1 predicted genes include single-exon genes and genes suspected to be incomplete (described in detail below). The areas selected for prediction from genome sequence are the areas flanking the retained stage 1 genes, as well as loci where alternative splice forms may exist. These steps, and the motivation for them, are discussed in more detail in the following paragraphs, and Figure 1, sections D, E and F give examples and summarise this process.

Where an assembled RNA-seq transcript, aligned relative to the genome sequence, overlaps another assembled transcript on the opposite strand, the transcript’s predicted UTR can contain all or part of the coding sequence from the adjacent transcript on the opposite strand. In stage 1, genes are predicted in a single direction in a single transcript, that is, although multiple genes are permitted to be predicted in a single transcript, they must all be in the same direction. As a result, where prediction from transcript sequences is carried out on UTR regions containing coding sequence on the opposite strand, we have observed a tendency to predict small false-positive single-exon genes (see Figure 1, section Div). This is because the reverse-complement of a coding sequence has a slightly higher G:C content and contains fewer stop codons than typically occur in UTRs, therefore these regions are often a closer match to the coding sequence model. This problem occurs even more frequently when unstranded RNA-seq is used and adjacent transcripts on opposite strands are assembled into a single locus. To correct this, all single-exon genes from stage 1 are discarded and predictions from genome sequence are carried out in those regions. Although single-exon genes are used for training, this is restricted to coding sequences over 600 nucleotides so that these small false-positives do not contaminate the training set. When genes are predicted from genome sequence in step 2, the prediction is allowed to be on either strand and these false-positive predictions therefore do not occur, leading to better results.

Transcript assemblies are likely to contain some low coverage, incomplete transcripts (Figure 1, section Bv). Attempting to predict a complete gene from an incomplete transcript sequence can lead to errors due to absent start or stop codons (Figure 1, section Dv). If the transcript is incomplete at the 3′ end and the gene’s stop codon is outside the transcript sequence then it is likely that no gene will be predicted from the transcript. If the 5′ end of the transcript is incomplete then the predicted gene will have an incorrect start codon, or be completely missed (Figure 1, section Dv). In these circumstances, a prediction from genome sequence is likely to be more accurate. Where the open reading frame of a coding sequence predicted in stage 1 can be extended beyond the bounds of the supporting assembled transcript, there is the possibility that the assembled transcript sequence and resulting predicted coding sequence are incomplete at the 5′ end. Such genes are therefore identified as genes that are suspected to be incomplete. Therefore, the stage 1 prediction is removed and stage 2 genome-based predictions are then carried out (Figure 1, sections D-Fv). Any intron sites supported by the partial transcripts will restrict the location of introns predicted in step 2 and the gene prediction is thus operating as an RNA-seq informed predictor, rather than completely *ab initio*.

In an effort to identify alternate splicing during stage 2, if the removal of an intron can extend an ORF across it without terminating at a stop codon, additional predictions from genome sequence in stage 2 are allowed in these regions (Figure 1, sections D-Fiii). This process allows correct predictions to be made where a transcript has been assembled with a false-positive intron, or where an alternatively spliced transcript retaining the intron sequence was not included in the transcript assembly, possibly due to low RNA-seq abundance.

In addition to correcting some of the inaccuracies in gene prediction from transcript sequences, prediction from genome sequence allows *ab initio* prediction of any genes that were not expressed under the experimental conditions used (Figure 1, sections ii and vii). Gene predictions of this kind are *ab initio* and therefore subject to greater uncertainties. In light of this, the final outputs of CodingQuarry make note of whether a final gene prediction was derived from transcript (stage 1) or genome-based (stage 2) prediction processes.

#### Post prediction filtering

The final stage of annotation that CodingQuarry carries out is the removal of genes likely to be false-positive predictions. Any gene with a coding sequence that translates to less than 30 amino acids is removed from the annotation. Where alternative splice variants are predicted, only variants with at least one unique intron, or 10 or more unique amino acids are retained. Finally, any gene predicted overlapping a larger gene on the opposite strand is removed where less than 20% of its coding sequence lies outside the bounds of the larger gene. As discussed earlier, false-positive predictions of this kind a common where transcripts overlap one another. While nested genes of this kind are known to occur, they are considered to be rare [51].

#### Gene discovery

Often one of the key interests of RNA-seq studies for annotation purposes is to discover previously unannotated genes in areas with evidence of transcription. For example, laterally transferred genes, which are of high relevance in fungal genomics [52,53], may be missed in homology or GHMM-based predictions due to a lack of homologs in closely related species or atypical codon usage patterns. To assist in this process, CodingQuarry forces a gene prediction in transcripts that have no overlapping gene prediction after the complete annotation run. This uses the same hidden Markov model as in stage 1, however the probability of a state transition to a non-coding transcript state is set to zero. These genes are not intended to be included in the main set of predicted annotations and are output separately as a set of “dubious” genes. Further efforts to verify which of these genes are genuine could include searches for pfam/anti-fam domains [54,55], blast searches to databases or experimental verification. However, this set is certain to contain a high proportion of false-positive genes, in part due to open reading frames occurring by chance within non-coding transcripts.

#### Merged transcripts

One of the final outputs of CodingQuarry reports the IDs of assembled transcripts suspected to be instances of transcripts merged in assembly due to overlapping UTRs. This output is based on the genes predicted by CodingQuarry, and reports the number and DNA strand orientations of the theoretical constituent transcripts. Reporting the orientation is important for unstranded RNA-seq data, where instances of sense-antisense (S-AS) overlap between UTRs can lead to transcripts on opposite strands assembling into single loci.

### Training and running other gene predictors for benchmarking

Comparisons were made with AUGUSTUS [9,10,14], and TransDecoder [56]. AUGUSTUS (using hints) and TransDecoder both leverage RNA-seq data and as such have comparable features with CodingQuarry. Though GeneMark-ET also uses RNA-seq data to assist annotation, comparisons were not possible at the time of submission due to its application to fungi being under development. It is important to note that GeneMark-ET uses RNA-seq data to assist in automated training, rather than to also subsequently inform and influence predictions.

AUGUSTUS was trained using the online training server [57] taking a FASTA file of assembled transcripts (in this case from TopHat-aligned RNA-seq read coverage generated by Cufflinks) and the genome sequence as input. This pipeline uses PASA [17] to generate a training set of genes from the transcript data, aligns the transcripts to the genome and uses hints generated from the alignment to assist in gene prediction. This pipeline does not train an untranslated region (UTR) model from assembled RNA-seq transcripts. Intron hints were also generated directly from the read to genome alignment generated by Tophat, however predicting with the hints produced by the training server produced predictions with better sensitivity and specificity when compared to the accepted annotations, and these results were therefore used for comparisons with CodingQuarry.

TransDecoder predicts genes from transcript sequences and uses the transcript-to-genome alignment to place predictions on the genome. Pfam domain searches are also used by TransDecoder to support gene predictions. TransDecoder was run using the TopHat/Cufflinks transcript assembly as per the instructions on the cited web page [56].

### Quantifying gene prediction accuracy

Measures of nucleotide, exon, intron and gene sensitivity and specificity, as described by Burset and Guigo [58], were used to compare the high-confidence sets with the various predictions. Sensitivity is the proportion of a given feature (nucleotides/exons/introns/genes) in the high-confidence set that are correctly predicted. Specificity is the proportion of features in the predicted set that are correct (i.e. exactly match the high-confidence set). A correct nucleotide prediction was defined to be a nucleotide within a predicted coding region that is also within a coding region of the high-confidence set. An incorrect nucleotide prediction was defined to be a nucleotide within a predicted coding region that is within an intron or intergenic region in the high-confidence set. A correct exon/intron was defined to be where the exon/intron boundaries in the predicted set were an exact match to the exon/intron boundaries in the high-confidence set. An incorrect exon/intron was defined to be where the exon/intron boundaries in the predicted set did not exactly match one of the exons/introns in the high-confidence set. A gene was defined to be correctly predicted if the gene was exactly the same as in the high-confidence set, and incorrect if the high-confidence set did not contain gene that matched exactly.

Where comparisons were made with the full set of genes in the annotation, all genes in the prediction and in the annotation were used to calculate the values of sensitivity and specificity. Where comparisons were made with the high-confidence annotation subsets, the region over which each of these values were calculated was bounded by the high-confidence set gene boundaries, and any overlapping gene in the predicted set.

## Results and discussion

Sensitivity and specificity values were calculated at the nucleotide, exon, intron and gene-level for CodingQuarry predictions and predictions made by TransDecoder and AUGUSTUS. Comparisons were made between predictions and high-confidence subsets (Table 1), and the full sets (Table 2) of *Sc. pombe* and *S. cerevisiae* gene annotations. CodingQuarry can be seen to outperform the other gene predictors in many of the measures. Impressively, CodingQuarry achieved a ~90% gene-level sensitivity when comparing predictions with the high-confidence subsets. This means that CodingQuarry predicts around 90% of the high-confidence set genes perfectly, which is around 4-5% more than the next best gene-level sensitivity result, belonging to AUGUSTUS (with hints), and around 10% better than TransDecoder, which also makes predictions from transcript sequences.

**Table 1:** **Comparisons between predictions and high-confidence gene sets for** ***Sc. pombe*** **and** ***S. cerevisiae***

	**Nucleotide**		**Exon**		**Intron**		**Gene**	
	**Sn**	**Sp**	**Sn**	**Sp**	**Sn**	**Sp**	**Sn**	**Sp**
*Sc. pombe (1898/5124 genes in high-confidence set)*								
CodingQuarry	**99.3**	**99.7**	**93.4**	**93.6**	94.5	96.7	**91.3**	**89.0**
AUGUSTUS	99.2	99.1	92.0	91.4	**95.7**	92.6	86.9	88.9
TransDecoder	95.4	99.3	84.5	86.3	88.5	**97.0**	80.2	73.5
*S. cerevisiae (5206/6575 genes in high-confidence set)*								
CodingQuarry	**99.2**	**99.8**	**90.0**	90.0	**79.0**	67.6	**90.4**	91.1
AUGUSTUS	97.5	99.7	84.7	**90.9**	74.4	**77.0**	85.0	**91.5**
TransDecoder	92.2	99.5	79.9	74.8	73.9	67.4	80.1	68.0

Sensitivity (Sn) is the proportion of a given feature (nucleotides/exons/introns/genes) in the high-confidence set that are correctly predicted. Specificity (Sp) is the proportion of features in the predicted set that are correct. Sensitivity and specificity calculations for nucleotides are made on nucleotides within coding regions. Further descriptions of these measures are given in the Implementation subsection titled “Quantifying prediction results”. The highest scores in each column for *Sc. pombe* and *S. cerevisiae* are shown in boldface.

**Table 2:** **Whole-genome comparisons between predictions and current** ***Sc. pombe*** **and** ***S. cerevisiae annotations***

	**Nucleotide**		**Exon**		**Intron**		**Gene**	
	**Sn**	**Sp**	**Sn**	**Sp**	**Sn**	**Sp**	**Sn**	**Sp**
*Sc. pombe (all 5124 genes)*								
CodingQuarry	**98.6**	98.9	**90.3**	89.4	92.6	95.2	**87.5**	83.0
AUGUSTUS	98.0	**99.3**	89.0	**90.6**	**94.2**	92.7	83.1	**87.7**
TransDecoder	93.4	99.2	80.8	85.4	85.3	**96.6**	76.3	72.5
*S. cerevisiae (all 6575 genes)*								
CodingQuarry	**97.2**	99.5	**76.1**	87.2	**64.4**	65.8	**76.6**	88.3
AUGUSTUS	95.4	99.6	71.1	**88.9**	60.5	**69.3**	71.5	**89.8**
TransDecoder	87.8	**99.7**	67.1	75.0	60.5	70.1	67.8	68.0

Sensitivity (Sn) is the proportion of a given feature (nucleotides/exons/introns/genes) in the annotation that are correctly predicted. Specificity (Sp) is the proportion of features in the predicted set that are correct. Sensitivity and specificity calculations for nucleotides are made on nucleotides within coding regions. Further descriptions of these measures are given in the Implementation subsection titled “Quantifying prediction results” The highest scores in each column for *Sc. pombe* and *S. cerevisiae* are shown in boldface.

An important consideration is that although both CodingQuarry and AUGUSTUS both use GHMMs, CodingQuarry operates very differently to AUGUSTUS. The main difference is that CodingQuarry combines predictions made initially from transcript sequences together with predictions from genome sequences. We assert that this is an important point in favour of CodingQuarry being considered for wider incorporation into automated annotation pipelines. Consensus between the predictions of different programs/tools can strengthen the confidence in the gene structure, particularly where genes are predicted by different methods. For example, CodingQuarry and AUGUSTUS predict 4,294 genes *Sc. pombe* genes identically, 95.0% of which exactly match the *Sc. pombe* annotation. In the case of *S. cerevisiae*, CodingQuarry and AUGUSTUS predict 4,813 genes identically, 95.4% of which are correct. This demonstrates that these subsets of genes have a higher specificity than either of the programs do individually, and can be considered higher confidence. If gene predictors operate in very similar ways, the fact that predictions agree is less significant.

The improved accuracy of CodingQuarry over alternative gene predictors is not achieved through protein homology-based prediction or refinement. Accurate gene predictions are therefore achievable when reliable sets of homologous proteins are not available. Such situations can arise when considering newly sequenced fungi, where closely related fungal species have not been sequenced or well annotated. However, if reliable homology evidence is available, CodingQuarry’s results have the potential to be further refined and improved by post-prediction annotation tools that merge predicted annotations with multiple sources of supporting evidence, such as EVidenceModeller [7] or Maker2 [19].

The closest competitor to CodingQuarry is AUGUSTUS, which derives all its gene predictions from genome sequences. However, when predicting genes from gene-dense genomes, the short intergenic distances make it possible for an intergenic region between two adjacent genes to be falsely annotated as an intron thus predicting a single merged gene where there should be two or more separate genes. We observed 32 and 25 instances of this in the AUGUSTUS predicted gene sets for *Sc. pombe* and *S. cerevisiae* respectively. When predicting directly from transcript sequences with CodingQuarry this is unlikely to occur, as introns are not predicted during stage 1 and adjacent genes would therefore need to be separated by an ORF to be falsely predicted as a single gene. As such, we see just one case of this error occurring in CodingQuarry predictions for *S. cerevisiae*, and two for *Sc. pombe*. This demonstrates an advantage to using CodingQuarry when annotating gene-dense fungal genomes. Notably, this advantage is also observed for TransDecoder, which also predicts from transcript sequences, with no cases of this error in the *S. cerevisiae* prediction and just one in *Sc. pombe*. However, TransDecoder achieved a much lower overall quality of prediction, with a ~10% lower sensitivity and ~10-20% lower specificity than CodingQuarry when compared to the high-confidence subsets and full sets of annotations (Tables 1 and 2). TransDecoder is intended to be used as part of a prediction pipeline and generates a set of genes to be used for training gene predictors. It is important to note that for its intended purpose, TransDecoder performs extremely well. However, based on the results shown in Tables 1 and 2, CodingQuarry was able to generate a larger and more accurate training set of genes.

**Figure 2:**
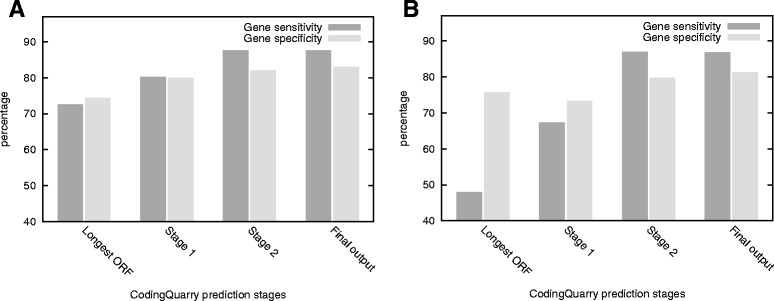
**Changes in CodingQuarry prediction accuracy at various stages of prediction of** ***Sc. pombe*** **genes.** The gene-level sensitivity and specificity is shown at various stages (See Figure 1 and Methods) within a CodingQuarry run. Results show comparisons with *Sc. pombe* where **A)** (left-hand panel) RNA-seq data strand information was used and **B)** (right-hand panel) strand information was ignored**.** Longest ORF is the initial training set, found by taking the longest open reading frame in each transcript to be a gene, stage 1 predictions are made from transcript sequences, stage 2 adds to and replaces some of stage 1 predictions by predicting from genome sequence. Filtering of likely false-positive genes (see Implementation section) takes place before a set of predicted genes is output as the “final output”. This output is the annotation generated by CodingQuarry.

As explained in the methods section, the predictions made by CodingQuarry are a combination of predictions from transcript sequences (stage 1), and predictions made from genome sequence (stage 2). A filtering step then removes genes likely to be false-positive predictions. The gene-level sensitivity and specificity of CodingQuarry, when compared to full *Sc. pombe* datasets, after each of these stages is displayed in Figure 2A. Figure 2A shows that the initial step of creating a training set using the longest ORF in each transcript has low values of sensitivity and specificity. An ~8% gene-level sensitivity and ~6% specificity improvement to predictions is made in stage 1, where these annotations are replaced by GHMM predicted genes. Part of the reason for this is that during stage 1, multiple genes predictions are allowed to be made within a single transcript, allowing a large number of genes residing in incorrectly “merged” transcripts to still be predicted. The second prediction stage again results in a jump in prediction accuracy, this time improving the gene-level sensitivity by ~8% and specificity by ~2%. This is due to the addition of genes predicted *ab initio* in regions without RNA-seq transcript coverage and the prediction of genes in regions where the transcript assembly is incomplete. Single-exon genes are also re-predicted stage 2. The final filtering step gives the final output CodingQuarry prediction. This step serves to improve specificity via the removal of false-positive genes, and therefore had little effect of the gene-level sensitivity (Figure 2A).

We observed variation in the accuracy of gene predictions made by all assessed gene predictors when comparing the results for *Sc. pombe* with those for *S. cerevisiae*. Fungal species have many complex differences relating to characteristics such as the number and size of introns [32], prevalence of alternative splicing [59], and gene density [60]. It is therefore reasonable to expect that gene prediction accuracy may vary across differing fungal species, and this can be seen in occurring in other published studies [15]. For predictions generated by CodingQuarry, a possible explanation is the contribution of RNA-seq evidence and how this could influence prediction accuracy. In the case of *Sc. pombe*, around 84% of the predicted genes result from a stage 1 transcript based predictions. However, the stage 1 component of the predicted genes is around 5% lower in S. cerevisiae. As these predictions RNA-seq driven, they are expected to be higher confidence, and it is therefore reasonable to expect the results to be better for *Sc. pombe* than *S. cerevisiae*.

Although stranded RNA-seq data is now readily available, a large quantity of non-stranded RNA-seq data is publically available. It is therefore important that CodingQuarry can deal with transcript assemblies resulting from either stranded or unstranded RNA-seq. Figure 2 shows gene-level sensitivity and specificity of *S. pombe* gene predictions made at stages within CodingQuarry with RNA-seq data where stranded information was ignored (Figure 2B), and where stranded information was included (Figure 2A). Gene level sensitivity and specificity for CodingQuarry’s final output predictions on *Sc. pombe* were less than 1% and 2% different between unstranded and stranded runs (respectively) (Figure 2). This result supports of the efficacy of CodingQuarry in overcoming issues in unstranded RNA-seq datasets. Comparisons between Figure 2A and B show that CodingQuarry predictions using the unstranded transcript assembly showed a ~25% improvement in gene level sensitivity going from stage 1 to stage 2 – further supporting the validity of the various processes employed in stage 2 to correct for annotation errors. We surmise that this is a direct result of sense-antisense (S-AS) transcript overlap resulting in merged transcripts composed of transcripts on opposite strands. This confounds prediction from transcript sequence, where genes are expected to be in the same direction as the transcript. As explained in the methods section, and evident from Figure 2A, this is corrected in stage 2 leading to comparable final outputs.

CodingQuarry reports on assembled transcripts which, according to the coding sequence predictions, may be multiple transcripts merged together in the assembly process. Where stranded RNA-seq is used, this is only a problem for overlapping transcripts on the same strand. For the *Sc. pombe* stranded RNA-seq experiment, there were 507 instances reported by CodingQuarry of likely transcript fusions. Of these, 64 were suspected to be the result of fusion of more than 2 transcripts. For *S. cerevisiae* there were more fusions detected: 1,060, with 452 of those suspected to result from the fusion of more than 2 transcripts. Given that different organisms of the same phyla can have very different gene densities and spacing, the higher number of fusions present in the *S. cerevisiae* transcript assembly is not surprising. Where transcripts are assembled from unstranded RNA-seq, there is the possibility of merged transcripts arising from S-AS transcript overlap. Although the splice sites in the transcript-to-genome alignment can help to separate these transcripts, it remains a problem where one or more of the transcripts align without introns. For *Sc. pombe*, the version of the transcript assembly generated without using strand information contained 1,219 instances where one transcript was suspected to be the fusion of multiple transcripts. 630 of these were suspected to be instances of transcripts fusions involving transcripts on opposite strands.

CodingQuarry has been designed for and tested on fungal genomes. It achieves a higher level of accuracy than competing methods by mixing predictions made from assembled transcript sequences with predictions made from assembled genome sequences. In theory, changes to the intron model used for prediction to allow the prediction of longer introns when predicting genes from assembled genome sequence would allow CodingQuarry to be applied to higher eukaryotes. However, in practice, the transcript assembly quality for RNA-seq datasets from higher eukaryotes does not result in enough correctly assembled full-length transcripts for this method to be advantageous. The limitations of transcript assembly quality to gene prediction have been previously noted [8]. Examples of factors contributing to this are the RNA-seq alignment/assembly being complicated by larger introns, and a higher prevalence of alternative splicing, as discussed in the Background section of this manuscript. It is therefore the opinion of the authors that it is unlikely that CodingQuarry would deliver similar improvement in genomes of higher eukaryotes as in fungal genomes, however this is something that may be explored in future studies.

CodingQuarry also outputs an additional set of “dubious” genes, as candidates for gene discovery. As described in the methods section, these genes are forced predictions in transcripts that, after running CodingQuarry steps 1 and 2, do not have an overlapping coding sequence prediction. 632 “dubious” genes are predicted for *Sc. pombe*, and 444 for *S. cerevisiae*. Of these, 25 and 16 overlap a gene in the annotation of *Sc. pombe* and *S. cerevisiae* respectively in the same coding frame. BLAST [61] was used to search for alignments between the protein sequences of dubious genes predictions with no coding sequence shared with genes in the annotation, and NCBI’s non-redundant database. Seven of these *Sc. pombe* genes aligned to an entry in nr with a protein level identity of 40% or better and e-value less than 10^−5^. Of these, six lay completely within a gene annotation on the opposite strand. For *S. cerevisiae,* 21 novel genes aligned to an entry in nr with a protein level identity of 40% or better and e-value better than 10^−5^*,* 10 of which lay completely within a annotated gene on the opposite strand. This result can either be viewed as the possibility of unannotated proteins in the test genome annotations, or, possible contamination of the nr database with translated sequences from non-coding RNA. We hope that this feature will assist researchers in gene discovery, however these predictions should be treated cautiously and we do not recommend their inclusion in a formal annotation dataset or submitted to databases without further validation.

## Conclusions

We have demonstrated the success of our method of using RNA-seq derived data in GHMMs for fungal gene prediction. For researchers studying the genomes of newly sequenced fungi, for which protein homology resources are absent or unreliable, CodingQuarry can be used as a single step in predicting protein-coding gene sequences with high accuracy. For more detailed annotation efforts, CodingQuarry offers an appropriate starting point for further refinement of annotations with additional supporting evidence.

## Availability and requirements

**Project name:** CodingQuarry.

**Project home page:**https://sourceforge.net/projects/codingquarry/.

**Operating system(s):** Platform independent.

**Programming language:** C++.

**Other requirements:** OpenMP.

**License:** GNU.

**Any restrictions to use by non-academics:** No.
